# A single immunization with H5N1 virus-like particle vaccine protects chickens against divergent H5N1 influenza viruses and vaccine efficacy is determined by adjuvant and dosage

**DOI:** 10.1080/22221751.2023.2287682

**Published:** 2023-11-23

**Authors:** Dexin Kong, Yanjuan He, Jiaxin Wang, Lanyan Chi, Xiang Ao, Hejia Ye, Weihong Qiu, Xiutong Zhu, Ming Liao, Huiying Fan

**Affiliations:** a College of Veterinary Medicine, South China Agricultural University, Guangzhou, People’s Republic of China; b Key Laboratory of Zoonosis Prevention and Control of Guangdong Province, Guangzhou, People’s Republic of China; c Key Laboratory of Veterinary Vaccine Innovation of the Ministry of Agriculture and Rural Affairs, Guangzhou, People’s Republic of China; d National and Regional Joint Engineering Laboratory for Medicament of Zoonosis Prevention and Control, Guangzhou, People’s Republic of China; e Guangzhou South China Biological Medicine Co., Ltd, Guangzhou, People’s Republic of China

**Keywords:** H5N1, virus-like particle, adjuvant, vaccine dose, neutralizing antibodies, cross-protection

## Abstract

The H5N1 subtype highly pathogenic avian influenza virus (HPAIV) reveals high variability and threatens poultry production and public health. To prevent the spread of H5N1 HPAIV, we developed an H5N1 virus-like particle (VLP) vaccine based on the insect cell-baculovirus expression system. Single immunization of the H5N1 VLP vaccines induced high levels of HI antibody titres and provided effective protection against homologous virus challenge comparable to the commercial inactivated vaccine. Meanwhile, we assessed the relative efﬁcacy of different adjuvants by carrying out a head-to-head comparison of the adjuvants ISA 201 and ISA 71 and evaluated whether the two adjuvants could induce broadly protective immunity. The ISA 71 adjuvanted vaccine induced significantly higher levels of Th1 and Th2 immune responses and provided superior cross-protection against antigenically divergent H5N1 virus challenge than the ISA 201 adjuvanted vaccine. Importantly, increasing the vaccine dose could further enhance the cross-protective efficacy of H5N1 VLP vaccine and confer completely sterilizing protection against antigenically divergent H5N1 virus challenge, which was mediated by neutralizing antibodies. Our results suggest that the H5N1 VLP vaccine can provide broad-spectrum protection against divergent H5N1 influenza viruses as determined by adjuvant and vaccine dose.

## Introduction

H5N1 HPAIV has been widely circulating in domestic poultry and wild birds around the world and is responsible for millions of domestic poultry deaths [1,2]. In addition to causing huge economic losses to the poultry industry worldwide, H5N1 HPAIV also poses a threat to public health. The H5N1 avian influenza virus (AIV) causes human infection without an intermediate host [3]. H5N1 AIV has continued to infect people worldwide since 2003, as of 14 July 2023, a total of 878 laboratory-confirmed cases of H5N1 AIV infection were reported to WHO, including 458 deaths [4]. Currently, H5N1 subtype HPAIV revealed a novel propensity after continuous evolution in nature, such as reassort with other influenza virus neuraminidase (NA) subtypes, including H5N6 and H5N8 [5,6]. This undoubtedly increases the risk of the H5 subtype HPAIV outbreak; thus preventing the H5N1 subtype HPAIV pandemic is imperative.

Vaccination is considered one of the current strategies to prevent and control the spread of H5N1 HPAIV in China [7]. However, the current commercial whole virus inactivated vaccines do not provide optimal protection against infection by novel H5N1 variants due to antigenic mismatch between vaccine and circulating virus stains. Besides, the production of the inactivated influenza vaccine relies on embryonated chicken eggs (ECEs) with many shortfalls including endogenous virus contamination, insufficient supply of ECEs during pandemic outbreaks, and biohazardous waste materials generation [7]. Meanwhile, H5N1 strains exhibit high variability and rapidly mutate making any vaccine strain unlikely to retain close identity with circulating stains. Therefore, it is particularly important to develop preferable alternatives to the traditional inactivated vaccine with optimal immunization strategies to provide cross-protection against H5N1 variants.

Virus-like particles (VLP) show high plasticity and scalability to develop multiple subtypes of influenza vaccines, including H5 [8], H6 [9], H7 [10], and H9 [11] subtypes, which are produced in various expression systems, such as insect cell-baculovirus [10], plant cells [12], and mammalian cells [13]. Furthermore, the innocuous VLP retains the structural and immunological properties of a native virus. Influenza VLP vaccines can induce comprehensive immune responses to provide cross-protection against homologous and heterologous influenza virus challenges by various administration routes [14,15]. Therefore, influenza VLP vaccine is considered one of the most promising alternatives to the traditional inactivated vaccine. Generally, influenza VLP constructs contain hemagglutinin (HA) and matrix protein M1. HA is the primary target antigen for eliciting serological immunity to the influenza virus [16]. HA is used to develop influenza subunit vaccines and has been shown to induce excellent protection against influenza virus infection in addition to the H5 serotype because H5 HA is a weak antigen [17,18].

Influenza vaccine efficacy is not only associated with HA similarity between vaccine and circulating virus strains but also involved antigen structure, adjuvant selection, vaccine antigen dose, and immunization strategy. Benefiting from the scalability of VLP, studies have shown that incorporating additional immunological proteins adjuvants such as nucleoprotein (NP), *Escherichia coli* heat-labile enterotoxin B subunit protein (LTB), and flagellin into influenza VLP induces better cross-protection against heterologous H5N1 challenges than VLP alone [19,20]. The strategies of multiple immunizations, sequential immunization, and combining with appropriate adjuvants are very major measures to improve the cross-protective efficacy of influenza vaccines against homologous, heterologous, and heterosubtypic influenza virus challenges [21-23]. Therefore, H5 influenza vaccines with optimal immunization strategies are particularly important for enhancing cross-protection efficacy.

In this study, we developed an H5N1 subtype VLP using a baculovirus expression vector system (BEVS). The immunogenicity of H5N1 VLP was evaluated by combining with two adjuvants, including MONTANIDETM ISA 201 VG (ISA 201) and MONTANIDETM ISA 71 VG (ISA 71), and using various antigen doses. Low-dose immunization of chickens with H5N1 VLP vaccines elicited robust HI antibody titres and induced protection against homologous H5N1 HPAIV challenge. ISA 71 adjuvanted VLP vaccine induced higher Th1-type and Th2-type immune responses and provided better cross-protection against antigenically divergent H5N1 HPAIV than ISA 201 adjuvanted VLP vaccine at the same dose. Enhancing doses of ISA 71 adjuvanted VLP vaccine provide complete protection against antigenically divergent H5N1 virus challenge in the absence of detectable challenge strains-specific HI antibody titres. Cross-protection efficacy correlates with neutralizing antibodies rather than HI. Our results suggest that the cross-protective ability of the H5N1 VLP vaccine is determined by adjuvant and vaccine dose, mediated by neutralizing antibodies.

## Materials and methods

### Ethics statement

All experiments with all available avian influenza A (H5N1) viruses were conducted in an animal biosafety level 3 laboratory and animal facility according to the protocols of South China Agricultural University (SCAU) (CNAS BL0011) protocols. All animals involved in the experiments were reviewed and approved by the Institution Animal Care and Use Committee at SCAU and treated by the guidelines (2017A002). At the end of the experiments, the discarded wastes and infected animal carcasses were autoclaved and incinerated to eliminate biohazards.

### Cells and viruses

*Spodoptera frugiperda* 9 (sf9) insect cells (Invitrogen, USA) were maintained in Sf-900 II serum-free medium (Gibco, USA) at 27°C. High Five ^TM^ (BTI-TN-5B1-4) suspension cells were routinely cultured in HF502C complete medium (Womei Bio, Suzhou, China) at 27°C in shaker flasks at a speed of 100–120 rpm. Madin-Darby canine kidney (MDCK) cells were maintained at 37°C in 5% CO_2_ in Dulbecco’s Modiﬁed Eagle’s Medium (DMEM) supplemented with 10% (v/v) heat-inactivated foetal bovine serum (Invitrogen, USA).

Two HPAI H5N1 viruses (SD57 and D889) originated from clade 2.3.2.1d (A/Chicken/Shandong/WFZC/2017(H5N1), H5N1-SD57) and clade 2.3.2.1e (A/Chicken/Guangdong/D889/2015(H5N1), H5N1-D889) were used for viral challenges (see Figure S1). All H5N1 viruses were propagated in 9- to 11-day-old specific-pathogen-free (SPF) embryonated chicken eggs at 37°C. Allantoic fluids were aliquoted and stored at −80°C until use. The 50% egg infectious dose (EID_50_) was calculated using the Reed-Muench method. The H5N1-SD57 virus was used as an H5 HA gene donor.

### Generation of recombinant baculovirus

To generate the VLP, the H5N1-SD57 virus was used as H5 HA gene donor; and the A/Chicken/Guangdong/16876/2016(H7N9) virus was used as M1 gene donor as described previously [10]. The HA and M1 genes were biochemically synthesized by BGI (Shenzhen, China). Genes of HA and M1 were codon-optimized for a high level of expression in High Five cells, and a 6×His epitope tag was simultaneously fused to the C-terminal end of the optimized gene. These two optimized genes were cloned into the pACEBac1 vector plasmid (Geneva Biotech, Geneva, Switzerland), respectively. The recombinant baculovirus was generated as described previously [10]. Briefly, the recombinant plasmids were transformed into *E. coli* DH10Bac to make recombinant bacmid baculovirus DNA, purified recombinant bacmid DNAs were transfected into sf9 insect cells using Cellfectin^TM^ II reagent (Invitrogen, Carlsbad, CA, USA) to obtain the recombinant baculovirus (rBV) in the culture supernatant. Following the manufacturer’s instructions, the recombinant baculoviruses were then amplified by infecting sf9 insect cells. All preparations of rBV were titrated using a rapid titration kit (BacPak Baculovirus Rapid Titer Kit; Clontech, Mountain View, CA, USA). The rBVs were designated as rBV-HA and rBV-M1, respectively.

### Indirect immunofluorescence assay

The indirect immunofluorescence assay (IFA) was carried out to detect the expression of HA and M1 protein in infected sf9 insect cells. The IFA was performed as following described. Briefly, sf9 insect cells were infected with rBV-HA or rBV-M1, respectively. After incubation for 48 h, the cells were fixed with pre-cooled methanol for 10 min at 4°C. The fixed cells were then incubated with the primary antibodies, including H5 subtype HA mouse monoclonal antibody against H5N1 HA protein (Zoonogen, Beijing, China) and His-tag mouse monoclonal antibody (Bioword, Nanjing, China) against M1 protein for 1 h at 37°C, respectively. After washing with phosphate-buﬀered saline (PBS, pH 7.2), the secondary antibody fluorescein (FITC)-conjugated goat anti-mouse IgG (H + L) (Bioword, Nanjing, China) was added to the cells and incubated at 37°C for 1 h. Fluorescent images were examined under an inverted ﬂuorescence microscope (Nikon, Ti-S, Japan).

### Preparation and purification of H5N1 VLP

H5N1 VLP was assembled in High Five cells. To generate the H5N1 VLP, High Five suspension cells were co-infected with the rBV-HA and rBV-M1 at a multiplicity of infection (MOI) of 2:1. After 4 days post-infection, cell culture supernatants were harvested and clarified by centrifugation (2000×*g* for 30 min at 4°C) followed by ultracentrifugation at 30,000×*g* for 60 min at 4°C. The sedimented particles were resuspended in PBS at 4°C overnight and further purified through a 20–30–45–60% discontinuous sucrose gradient at 100,000×*g* for 1 h at 4°C. The functionality of HA protein incorporated into VLP was quantified by hemagglutination assay (HA assay) using 1% (v/v) chicken red blood cells. The concentration of the H5N1 VLP was measured using the Pierce BCA Protein Assay Kit (Thermo Fisher Scientific).

### Electron microscopy

The obtained VLP suspension was adsorbed onto a carbon parlodion-coated copper grid for 2 min. Excess VLP suspension was removed by blotting with filter paper, and the grid was immediately stained with 1% phosphotungstic acid for 10 min. Excess stain was removed by filter paper, and the samples were examined using a transmission electron microscope (Talos L120C, FEI, Czech).

### SDS-PAGE and western blot

To determine the expression of HA and M1 protein, SDS-PAGE and western blot were performed as described previously [10]. Briefly, the protein samples were mixed with 5×SDS-PAGE loading buffer (Dingguo, Guangzhou, China) and boiled for 10 min, then separated by 10% Tris-Glycine gels, and stained using Coomassie Brilliant Blue (Dingguo, Guangzhou, China) for SDS-PAGE analysis. The protein bands were also transferred to nitrocellulose membranes (Bio-Rad, Guangzhou, China) for western blot analysis. The membranes were blocked with 5% (W/V) skim milk in PBST (PBS containing 0.05% (v/v) tween 20) for 1 h at room temperature. Membranes were subsequently incubated with H5 subtype HA mouse monoclonal antibody (Zoonogen, Beijing, China) and His-tag mouse monoclonal antibody (Bioword, Nanjing, China), respectively. The blots were then washed five times with PBST and incubated with a horseradish-peroxidase-conjugated goat anti-mouse IgG antibody (LI-COR, USA) for 1 h at room temperature. Finally, the proteins were visualized by chemiluminescence (LI-COR Odyssey).

### Vaccination and viral challenge

Three-week-old SPF chickens were purchased from the Experimental Animal Center (Xinxing Dahuanong Eggs Co., Ltd, Guangdong, China). They were maintained according to the South China Agricultural University's guidelines for the care and use of laboratory animals. H5N1 VLP was examined for immunogenicity and vaccination efficacy in three independent experiments (Exp 1, Exp 2, and Exp 3) as described in Supplementary Table 1. ISA 201 (Seppic, Paris, France) and ISA 71 (Seppic, Paris, France) were used as vaccine adjuvants. In Exp 1, as a homologous protection study, chickens (*N* = 10) were subcutaneously immunized once with 40 μg of H5N1 VLP (adjuvanted with ISA 201 or ISA 71), the inactivated reassortant avian influenza virus (H5 + H7) trivalent vaccine (composition H5N2 strains rSD57 and rFJ56, H7N9 strain rLN79) (South China Biological Medicine Co., Ltd, Guangzhou, China) immunized and PBS immunized group was used as a vaccine comparison control group and negative control group, respectively. 3 weeks after immunization, chickens were inoculated intranasally challenged with 10^6.0^ EID_50_ (0.2 ml) HPAI H5N1-SD57 virus. Exp 2 as an adjuvant comparison study, in Exp 2, chickens (*N* = 13) were subcutaneously immunized once with 40 μg of H5N1 VLP (adjuvanted with ISA 201 or ISA 71). Exp 3 as a dose escalation study, in Exp 3, chickens (*N* = 10) were subcutaneously immunized once with 60 μg or 80 μg of H5N1 VLP (adjuvanted with ISA 71). The negative controls for Exp 2 and Exp 3 were PBS-vaccinated chickens. The commercial vaccine was used as a vaccine comparison control in Exp 3. The vaccine was administered in a volume of 300 μl. 3 weeks after immunization, chickens of Exp 2 and Exp 3 were inoculated intranasally challenged with 10^6.0^ EID_50_ (0.2 ml) HPAI H5N1-D889 virus. Chickens were monitored for clinical signs and mortality for 14 days post-challenge (PC). All surviving chickens were killed humanely at the end of the monitoring experiments.

### Sample collection

Sera samples were collected at 3 weeks post-vaccination and stored in aliquots at −80°C until assayed. To evaluate the ability of vaccine candidates to induce immune responses and to further estimate the immune types, the PBMCs from each group (*N* = 3) in Exp 2 were isolated at 19 days after immunization and stimulated with H5N1-D889 virus or H5N1 VLP *in vitro*. To determine virus positivity or shedding from individual chickens, the oropharyngeal and cloacal swab samples were collected at 5 days post-challenge in Exp 1. The oropharyngeal and cloacal swab samples were collected at 3, 5, and 7 days post-challenge in Exp 2 and Exp 3. The swab samples were resuspended in 1 ml of PBS supplemented with 2000 mg/ml streptomycin, and 2000 IU/ml penicillin. The suspensions were centrifuged at 3000×*g* for 10 min, and 0.1 ml of the supernatants from the oropharyngeal or cloacal swabs were used to inoculate the allantoic cavities of 10-day-old SPF chicken embryos (3 eggs/sample). After incubation for 48 h at 37°C, the allantoic ﬂuids were tested for hemagglutination activity. A virus isolation positive swab means one or more of the inoculated egg allantoic fluids with reciprocal of the hemagglutination titres was higher than 4.

### Hemagglutination inhibition assay

Hemagglutination inhibition (HI) assay was performed using standard methods [24]. Brieﬂy, the chicken sera were serially diluted twofold and incubated with 4 HA units (HAU) of H5N1-SD57 virus or H5N1-D889 virus for 1 h at room temperature. Then 25 µl of a 1% suspension of chicken red blood cells (RBC) was added to each well and incubated at room temperature for 30 min. The HI titre was expressed as the reciprocal of the highest serum dilution that completely inhibited the hemagglutination.

### Virus neutralization assay

The neutralization assay was performed as described previously [10]. Briefly, MDCK cells were plated into 96-well plates. heat-inactivated serum samples were serially diluted with DMEM medium containing 2 mg/ml BSA (Dingguo, Guangzhou, China) and 0.5 μg/ml TPCK-trypsin (Dingguo, Guangzhou, China), and then mixed with equal volumes of 100 mean tissue culture infective dose (TCID_50_) of H5N1-D889 virus. After incubation at 37°C for 1 h, the mixtures of serum and virus were added to the MDCK cells in 96-well plates and cultured for 72 h. After 72 h of incubation, cell supernatants were harvested and transferred to V-bottom 96-wells plates. The presence of the virus was detected using a hemagglutination assay. Neutralizing antibody titres were defined as the reciprocal of the highest serum dilution that neutralized the virus in cell supernatants.

### Isolation and stimulation of chickens PBMCs

Peripheral blood mononuclear cells (PBMCs) were prepared for cytokine assays. PBMCs were isolated from peripheral blood using Ficoll-Hypaque density sedimentation (Tbdscience, Tianjin, China) according to the manufacturer’s instructions. After contaminating red blood cells (RBC) present in the isolated cells were lysed using RBC lysis buﬀer (Solarbio, China), and single cells were collected. PBMCs single-cell suspensions were cultured in complete Roswell Park Memorial Institute (RPMI) 1640 medium containing 10% FBS and 1% penicillin–streptomycin/L-glutamine (Gibco, Carlsbad, CA, USA) at a final concentration of 1 × 10^6^ cells/ml. Cells were stimulated with 10^3^ TCID_50_ of H5N1-D889 virus or 20 μg of H5N1 VLP and incubated for 8 h at 37°C. Then, cells were harvested for RNA extraction. Cytokine expression levels of cells were evaluated using qRT-PCR.

### Cytokine assays using quantitative real-time PCR (qRT-PCR)

Total mRNA was extracted using total RNA extraction kits (Feijie, Shanghai, China); 500 ng of total mRNA was converted into cDNA using HiScript Reverse Transcriptase (Vazyme, Nanjing, China) according to the manufacturer’s instructions. mRNA expressions were examined using qRT-PCR with ChamQ Universal SYBR qPCR master mix (Vazyme, Nanjing, China) using a Bio-Rad CFX Applied System PCR instrument (Bio-Rad Laboratories Inc., Hercules, CA). Sequences of primers used for qRT-PCR are shown in Supplementary Table 2. The analysed speciﬁc gene level was normalized with a housekeeping gene β-actin of the respective treatment group and results were expressed in fold change.

### Statistical analysis

Experimental data are presented as mean ± SD of the mean. GraphPad Prism 9 software was used for data analysis. The statistical analyses of HI antibody titres and cytokine levels were performed using one-way analysis of variance or an unpaired *t*-test. The statistical analyses of neutralizing antibody titres were performed using Mann–Whitney *U* test. The survival status after the challenge was analysed using Log-rank (Mantel–Cox) test. Signiﬁcant differences are denoted by *(*P* < 0.05), ** (*P* < 0.01), *** (*P* < 0.001), or **** (*P* < 0.0001).

## Results

### Production and characterization of H5N1 VLP

**Figure 1.**
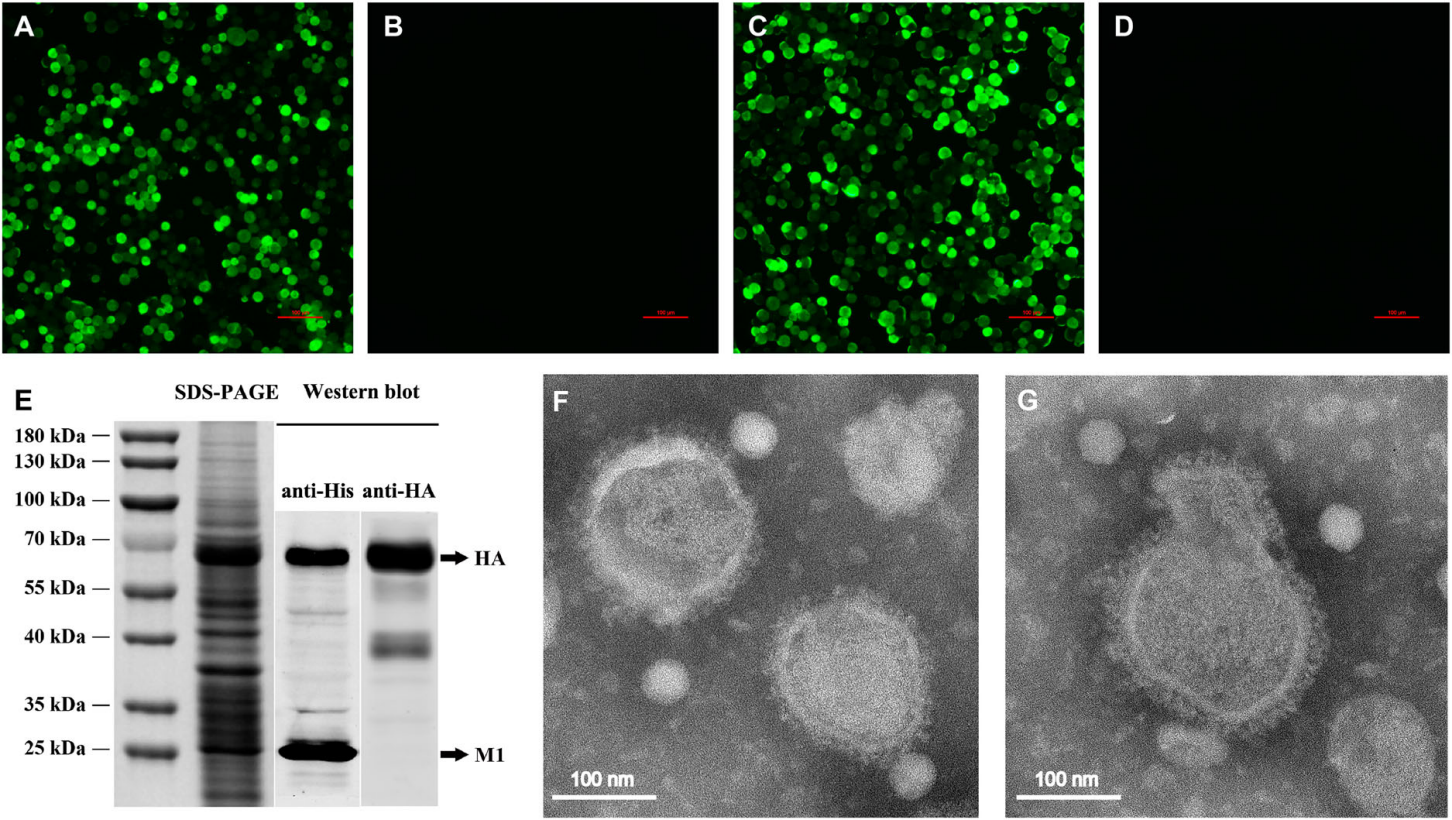
Production and characterization of H5N1 VLP. The expression of the HA and M1 proteins was observed in the infected sf9 cells using IFA. Sf9 cells infected with rBV-HA (A), rBV-M1 (C), or only empty baculoviruses (B), (D) after 48 h. (E) The expression of the HA and M1 proteins on the VLP was analysed using SDS-PAGE gels with coomassie blue staining and validated by western blot using the His-tag mouse monoclonal antibody and H5 subtype HA mouse monoclonal antibody. (F), (G) Negative staining electron microscopy of the H5N1 VLP. Puriﬁed VLPs were stained using 1% phosphotungstic acid.

The HA gene of the H5N1 subtype and the M1 gene of the H7N9 subtype were transformed into the bacmid DNA. The recombinant baculoviruses expressing the HA and M1 genes were generated by transfecting sf9 cells with recombinant bacmid. The specific fluorescence of the HA and M1 proteins was observed in the infected sf9 cells using IFA (Figure 1A, C), whereas there was no specific fluorescence in the control baculovirus-infected cells (Figure 1B, D), indicating successful expression of HA and M1 proteins. Subsequently, the H5N1 VLP was produced in High Five cells by co-infection of the rBVs expressing HA and M1. The production of VLP was confirmed using SDS-PAGE and western blotting (Figure 1E). The molecular mass of HA and M1 proteins was ∼65 and ∼28 kDa, respectively. The hemagglutination activity of the H5N1 VLP reached 2^13^. The size and morphology of H5N1 VLP were examined by transmission electron microscopy (Figure 1F, G). The average size of the VLP was 100 nm; the morphology of the VLP resembles that of influenza virus particles, and the spikes were observed on spherical surfaces which mimic influenza virus HA protein on the native virions. These results showed that the H5N1 VLP was successfully assembled, with similar morphology and size to natural influenza virions.

### Immunization of H5N1 VLP vaccines induces serum antibodies against homologous H5N1 virus challenge

**Figure 2.**
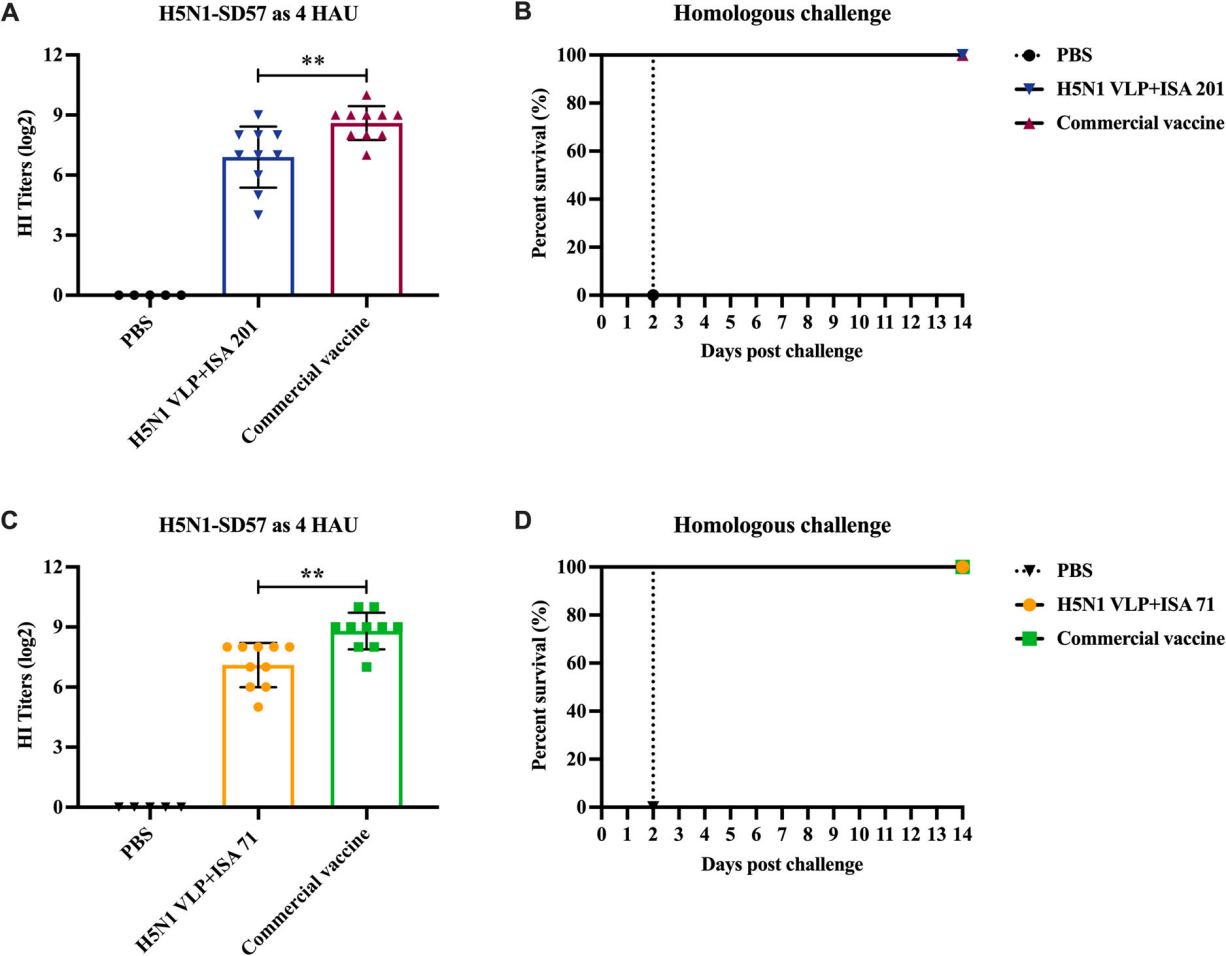
Hemagglutinin inhibition (HI) titres of SPF chickens after immunization and survival rates of chickens after challenge. Chickens were subcutaneously immunized with 40 μg of H5N1 VLP vaccine (adjuvanted with ISA 201 or ISA 71) or commercial vaccine. (A, C) The serum samples from each group of vaccinated chickens were collected at 3 weeks post-vaccination, HI titres against H5N1-SD57 virus. The HI titres among vaccination groups were compared using an unpaired *t*-test. Statistically significant differences are indicated by **p < 0.01. (B, D) At 3 weeks post-vaccination, groups of chickens were intranasally challenged with 10^6.0^ EID_50_ of the homologous H5N1-SD57 virus. Survival rates of chickens were measured daily for 14 days after challenge. PBS group (*n* = 5), vaccine group (*n* = 10).

To examine the capacity of the H5N1 VLP vaccine, SPF chickens were immunized with 40 μg of H5N1 VLP vaccine (adjuvanted with ISA 201 or ISA 71) and commercial inactivated vaccine, respectively. The serum HI antibody titres against the homologous virus were measured by HI assay at 3 weeks after a single-dose vaccination. The results demonstrated that the commercial vaccine induced significantly higher HI antibody titres than the ISA 201 and ISA 71 adjuvanted H5N1 VLP vaccines (Figure 2A, C). Furthermore, similar levels of HI antibody titres were observed in chickens receiving H5N1 VLP vaccines adjuvanted with ISA 201 (6.9 log2) or ISA 71 (7.1 log2). The results show that both the ISA 201 and ISA 71 adjuvanted H5N1 VLP vaccines induce effective antibody response in chickens.

To evaluate the protective efficacy of the vaccine, all immunized chickens were challenged with the homologous H5N1-SD57 virus. The survival rates and morbidity of chickens in each group were monitored for 14 days after the challenge. After a lethal virus challenge, the chickens receiving PBS rapidly died with typical clinical signs (Figure 2B, D). In contrast, all chickens survived in the H5N1 VLP vaccine (adjuvanted with ISA 201 or ISA 71) and commercial vaccine groups, without showing any clinical signs during 14 days of the monitoring period (Figure 2B, D). Oropharynx and cloaca swabs were collected on day 5 post-challenge to monitor virus shedding. No swabs were taken from the mock chickens after the challenge because they all died at the sampling time. Virus shedding was not detected in chickens from both the ISA 201 and ISA 71 adjuvanted H5N1 VLP vaccine groups, and similarly, chickens from commercial vaccine groups were free from virus detection (see Supplementary Table 3). Collectively, these results indicate that both the ISA 201 and ISA 71 adjuvanted H5N1 VLP vaccines can protect chickens from lethal challenge with the homologous HPAI H5N1 virus comparable to the commercial vaccine.

### ISA 71 adjuvant enhances Th1-type and Th2-type immune responses of H5N1 VLP vaccine

**Figure 3.**
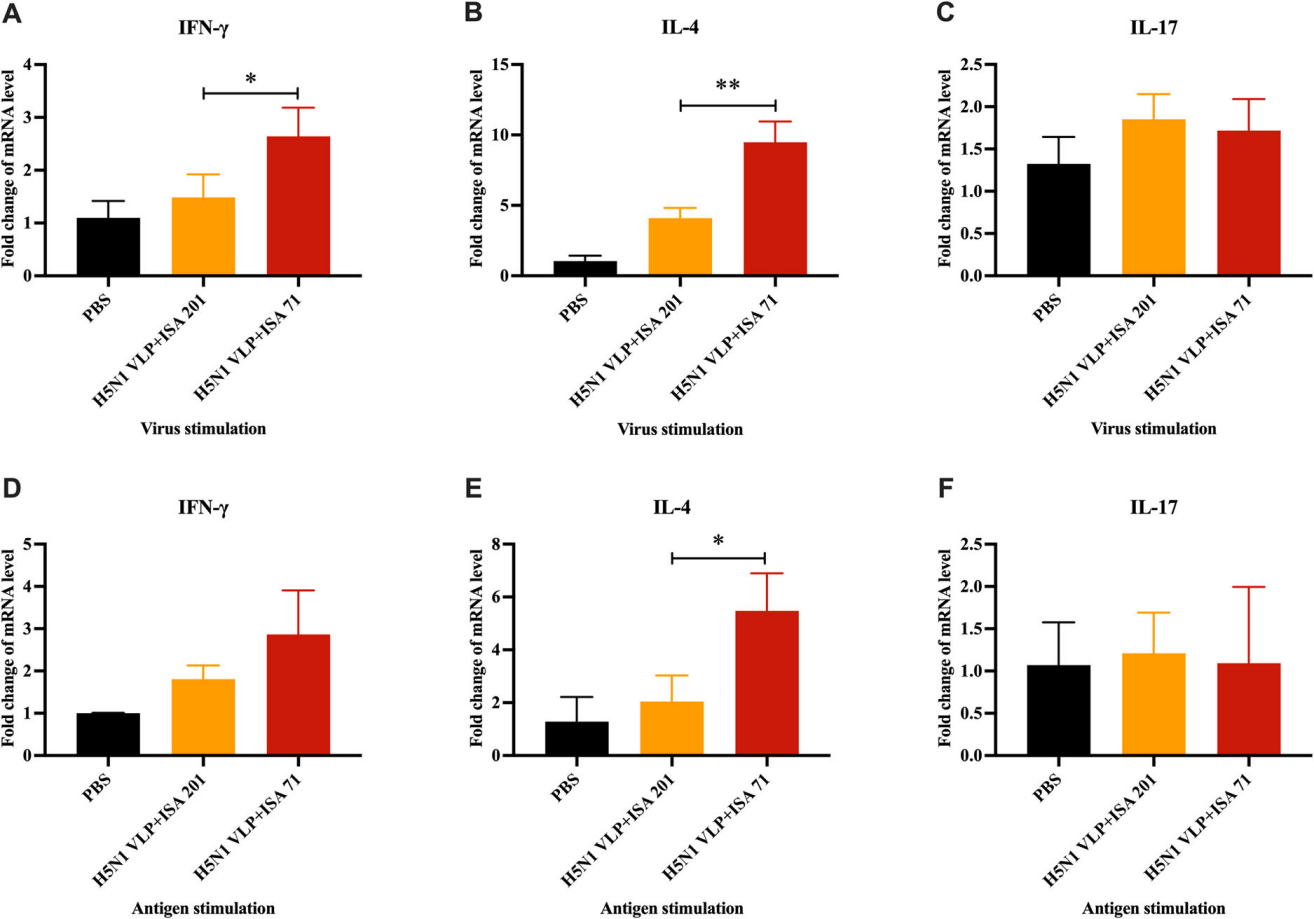
Cytokine expression levels in PBMCs of SPF chickens (*n* = 3). The mRNA expression levels of IFN-γ, IL-4, and IL-17 after virus stimulation (A, B, C) or antigen stimulation (D, E, F) were determined using qRT-PCR. The cytokine levels among groups were compared using one-way ANOVA followed by Tukey’s multiple comparison test. Data represented mean ± SD. Statistical significance of differences is illustrated as follows: *p < 0.05, **p < 0.01.

**Figure 4.**
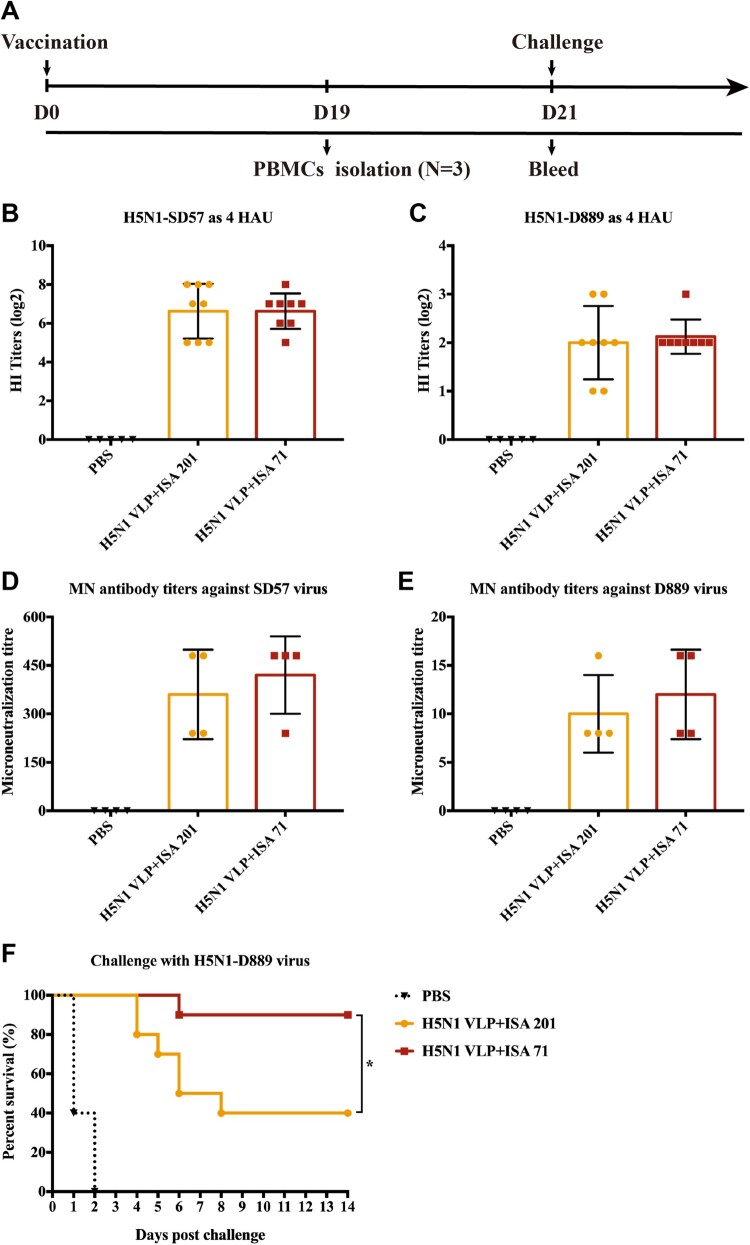
Immunogenicity and protection against HPAI H5N1-D889 virus. (A) Animal experimental 2 design for immunization and challenge. The serum samples from each group of vaccinated chickens were collected at 3 weeks post-vaccination. (B) HI titres against H5N1-SD57 virus. (C) HI titres against H5N1-D889 virus. Neutralizing antibodies from each group (*n* = 4) were measured by neutralization assay, (D) Neutralizing antibodies titres against 100 TCID_50_ of H5N1-SD57 virus. (E) Neutralizing antibodies titres against 100 TCID_50_ of H5N1-D889 virus. (F) Groups of chickens were intranasally challenged with 10^6.0^ EID_50_ of the antigenically divergent H5N1-D889 virus. Survival rates of the chickens after challenge. PBS group (*n* = 5), vaccine group (*n* = 10). *p < 0.05 by Log-rank (Mantel–Cox) test.

To evaluate the effect of adjuvant on the immune response types, the level of the cytokines IFN-γ, IL-4, and IL-17, associated with Th1-type, Th2-type, and Th17-type immune responses, respectively, were determined. SPF chickens were immunized with H5N1 VLP with ISA 201 or ISA 71, and PBMCs were isolated at 19 days after immunization and stimulated with H5N1-D889 virus or H5N1 VLP *in vitro* (Figure 4A). For virus stimulation, the results indicated that chickens receiving ISA 71 adjuvanted H5N1 VLP vaccine had significantly higher IFN-γ and IL-4 mRNA expression levels than chickens receiving ISA 201 adjuvanted H5N1 VLP vaccine (Figure 3A, B). Similarly, after antigen stimulation, chickens receiving ISA 71 adjuvanted H5N1 VLP vaccine also had higher mRNA expression levels of IFN-γ and IL-4 than chickens receiving ISA 201 adjuvant H5N1 VLP vaccine (Figure 3D, E). The mRNA levels of IL-17 were not significantly different in vaccinated chickens, whether stimulated by antigen or by virus (Figure 3C, F). Meanwhile, the results also showed that IL-4 expression levels in vaccinated chickens stimulated by virus were significantly higher than that stimulated by antigen (see Figure S2). Overall, these results indicate that ISA 71 adjuvant augments Th1-type and Th2-type immune responses of H5N1 VLP vaccine compared to ISA 201 adjuvant.

### ISA 71 adjuvanted H5N1 VLP vaccine offers better cross-protection against antigenically divergent H5N1 virus challenge

To evaluate the effect of adjuvant for the cross-protective efficacy of H5N1 VLP vaccine, SPF chickens were immunized with 40 μg of H5N1 VLP adjuvanted with ISA 201 or ISA 71, respectively. Serum samples were collected at 3 weeks post-vaccination to evaluate the cross-reactivity (Figure 4A). HI and microneutralization (MN) antibodies were measured against homologous H5N1-SD57 virus and antigenically divergent H5N1-D889 virus. Compared to chickens immunized with ISA 201 adjuvanted H5N1 VLP vaccine, chickens receiving H5N1 VLP with ISA 71 generally induced comparable HI titres against H5N1-SD57 virus and H5N1-D889 virus (Figure 4B, C). However, the ISA 71 adjuvanted H5N1 VLP vaccine induced slightly higher MN antibody titres against H5N1-SD57 virus and H5N1-D889 virus than the ISA 201 adjuvanted H5N1 VLP vaccine (Figure 4D, E). Our results suggest that both the ISA 201 and ISA 71 adjuvanted H5N1 VLP vaccines induce high HI and MN antibody titres against homologous H5N1-SD57 virus, but induced only low levels of HI and MN antibody titres against antigenically divergent H5N1-D889 virus.

To evaluate the cross-protective efficacy of the vaccine, each group of immunized chickens (*N* = 10) was challenged with the antigenically divergent H5N1-D889 virus. After a lethal virus challenge, 90% (9/10) of chickens survived in the ISA 71 adjuvanted H5N1 VLP vaccine group. In contrast, only 40% (4/10) of chickens survived in the ISA 201 adjuvanted H5N1 VLP vaccine group, and all chickens died in the PBS control group (Figure 4F). Oropharynx and cloaca swabs were collected at 3, 5, and 7 days post-challenge to monitor virus shedding. No swabs were taken from dead chickens after the challenge. Seven chickens in the ISA 71 adjuvanted H5N1 VLP vaccine group and six chickens in the ISA 201 adjuvanted H5N1 VLP vaccine group recovered viruses from oropharynx and cloacal swab samples (Table 1). Taken together, these findings indicate that ISA 71 is a more potential adjuvant for H5N1 VLP vaccines than ISA 201.

**Table 1.** Virus shedding after H5N1-D889 virus challenge of chickens.

Group	Challenge virus	Oropharyngeal swab (virus shedding number/total number)			Cloacal swab (virus shedding number/total number)			Total virus shedding number/total number
		3 dpc^a^	5 dpc	7 dpc	3 dpc	5 dpc	7 dpc	
H5N1VLP + ISA 201	D889	2/10	2/7	1/5	3/10	1/7	2/5	6/10
H5N1VLP + ISA 71	D889	0/10	1/10	0/9	1/10	3/10	6/9	7/10
PBS	D889	NA^b^	NA	NA	NA	NA	NA	NA

D889 is the virus of A/Chicken/Guangdong/D889/2015(H5N1). The oropharyngeal and cloacal swab samples were collected at 3, 5, and 7 days post-challenge. Virus positivity or shedding was determined by inoculating each swab solution into 3 eggs of 10-day-old specific-pathogen-free chicken embryos. ^a^dpc, days post-challenge. ^b^NA, not applicable due to the death of chickens.

### Enhanced doses of H5N1 VLP vaccine induce higher levels of HI and MN antibodies

**Figure 5.**
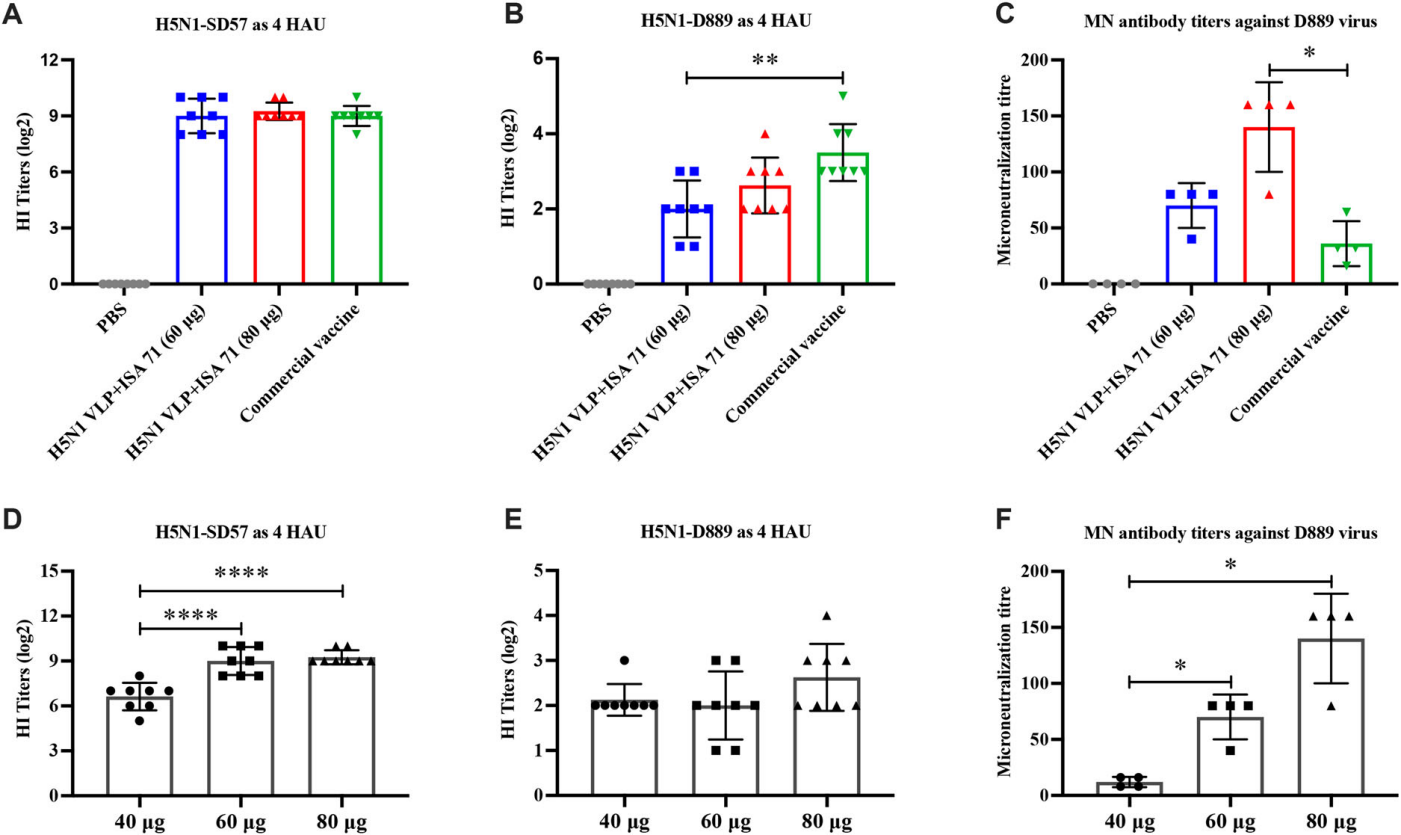
HI and neutralizing antibody titres of serum from vaccinated chickens. The serum samples from each group of vaccinated chickens were collected at 3 weeks post-vaccination. (A) HI titres against H5N1-SD57 virus. (B) HI titres against H5N1-D889 virus. (C) Neutralizing antibodies from each group (*n* = 4) were measured by neutralization assay, neutralizing antibodies titres against 100 TCID_50_ of H5N1-D889 virus. (D) HI titres against H5N1-SD57 virus induced by 40, 60, and 80 μg of adjuvanted H5N1 VLP vaccine. (E) HI titres against H5N1-D889 virus induced by 40, 60, and 80 μg of adjuvanted H5N1 VLP vaccine. (F) Neutralizing antibodies titres against H5N1-D889 virus induced by 40, 60, and 80 μg of adjuvanted H5N1 VLP vaccine. PBS group (*n* = 5), vaccine group (*n* = 10). The HI antibody titres among vaccination groups were compared using one-way ANOVA followed by Tukey’s multiple-comparison test. The neutralizing antibody titres among vaccination groups were compared using Mann–Whitney *U* test. Statistically significant differences are indicated by *p < 0.05, **p < 0.01, or ****p < 0.0001.

To evaluate the effect of antigen dose on the cross-protective efficacy of H5N1 VLP vaccine, SPF chickens were immunized with 60 μg or 80 μg of H5N1 VLP adjuvanted with ISA 71, and commercial vaccine as a control. Serum samples were collected at 3 weeks post-vaccination and HI and MN antibodies were measured. Both the 60 and 80 μg of H5N1 VLP vaccines induced high levels of HI antibody titres against homologous H5N1-SD57 virus, with no significant difference compared with commercial vaccine (Figure 5A). Both the H5N1 VLP vaccines induced low HI antibody titres against antigenically divergent H5N1-D889 virus (Figure 5B). Besides, the mean HI titre against H5N1-D889 virus induced by the commercial vaccine was 3.5 log2, which was significantly higher than that induced by the 60 μg of H5N1 VLP vaccine (2 log2). Serum-neutralization activity against the H5N1-D889 virus was determined. The results showed that the 80 μg of H5N1 VLP vaccine induced significantly higher MN antibody titres than the commercial vaccine (Figure 5C).

The dose effect was evaluated by comparing the differences in serum antibody titres induced by three doses of H5N1 VLP vaccine. The HI titres against homologous H5N1-SD57 virus induced by the 60 μg or 80 μg of H5N1 VLP vaccine were significantly higher than those induced by the 40 μg of H5N1 VLP vaccine (Figure 5D). The HI titres against antigenically divergent H5N1-D889 virus were not significantly different among the 40, 60, and 80 μg doses of the H5N1 VLP vaccine (Figure 5E). The neutralizing antibody responses were antigen dependent, and the MN antibody titres against H5N1-D889 virus induced by the high-dose H5N1 VLP vaccine were significantly higher than those induced by the low-dose H5N1 VLP vaccine (Figure 5F). These findings indicate that the augment of vaccine dose fails to induce effective HI antibody titres against antigenically divergent H5N1 virus, but improves neutralizing antibody titres against antigenically divergent H5N1 virus.

### High dose of H5N1 VLP vaccine confers full protection against antigenically divergent H5N1 virus challenge

**Figure 6.**
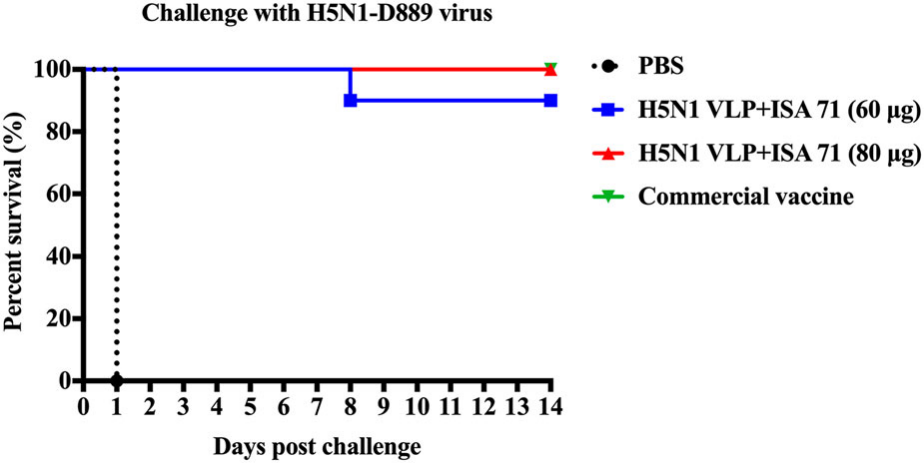
Survival rates of the chickens after lethal H5N1-D889 virus challenge. At 3 weeks after immunization, groups of immunized chickens were intranasally challenged with a high lethal dose (10^6.0^ EID_50_) of H5N1-D889 virus. Survival rates of chicken were measured daily for 14 days after challenge. PBS group (*n* = 5), vaccine group (*n* = 10).

To evaluate the cross-protection efficacy of increasing doses of H5N1 VLP vaccines, all immunized chickens were challenged with the antigenically divergent H5N1-D889 virus. Consistent with the antibody responses, chickens immunized with 80 μg of H5N1 VLP vaccine and commercial vaccine survived, without showing any clinical signs during 14 days of the monitoring period. In contrast, 90% (9/10) of chickens receiving 60 μg of H5N1 VLP vaccine survived, and all chickens died in the PBS control group (Figure 6).

Oropharynx and cloaca swabs were collected at 3, 5, and 7 days post-challenge to monitor virus shedding. No swabs were taken from dead chickens after the challenge. Virus shedding was not detected in chickens from the 80 μg of H5N1 VLP vaccine group. One chicken in the commercial vaccine group and two chickens in the 60 μg of H5N1 VLP vaccine group recovered viruses from oropharynx and cloacal swab samples (Table 2). Taken together, these findings indicate that the augment of H5N1 VLP vaccine dose improved cross-protection against challenges with antigenically divergent H5N1 virus and that protection capacity correlated with neutralizing antibodies.

**Table 2.** Virus shedding of chickens challenged with H5N1-D889 virus.

Group	Challenge virus	Oropharyngeal swab (virus shedding number/total number)			Cloacal swab (virus shedding number/total number)			Total virus shedding number/total number
		3 dpc^a^	5 dpc	7 dpc	3 dpc	5 dpc	7 dpc	
H5N1VLP + ISA 71 (60 µg)	D889	0/10	0/10	0/10	0/10	1/10	2/10	2/10
H5N1VLP + ISA 71 (80 µg)	D889	0/10	0/10	0/10	0/10	0/10	0/10	0/10
Commercial vaccine	D889	0/10	0/10	0/10	0/10	1/10	1/10	1/10
PBS	D889	NA^b^	NA	NA	NA	NA	NA	NA

Chickens were inoculated intranasally challenged with 10^6.0^ EID_50_ of H5N1-D889 virus. The oropharyngeal and cloacal swab samples were collected at 3, 5, and 7 days post-challenge. Virus positivity or shedding was determined by inoculating each swab solution into 3 eggs of 10-day-old specific-pathogen-free chicken embryos. ^a^dpc, days post-challenge. ^b^NA, not applicable due to the death of chickens.

## Discussion

H5N1 and H5Nx HPAI emerging variants threaten poultry industry, food security, and public health [25–27]. Vaccination is essential for preventing and controlling the spread of H5 subtype influenza virus. H5N1 HPAIV is highly variable in nature, and influenza vaccine efficacy is closely related to the matching of vaccine and circulating strains. Therefore, technologies that allow for timely update of strains and rapid production of vaccines are most advantageous during epidemics of emerging variants. In this regard, the technology of VLP production at a baculovirus expression vector system is undoubtedly qualified in response to emerging H5N1 viruses. It is accepted that influenza vaccine development based on BEVS has unique advantages, such as straightforward scale-up, excellent safety, and short production times [28]. Meanwhile, the baculovirus-derived influenza VLP vaccines have proved to be a safe and effective alternative to the traditional inactivated vaccine [29].

In this study, an H5N1 VLP was prepared in the BEVS and the efficacy of the H5N1 VLP vaccine was evaluated in chickens. In the homologous protection study, the ISA 201 and ISA 71 adjuvanted H5N1 VLP vaccines induced similar levels of HI antibody titres, which were significantly lower than those induced by the commercial vaccine. Both the H5N1 VLP vaccine (adjuvanted with ISA 201 or ISA 71) and commercial vaccine provided good protection against homologous H5N1 HPAIV challenge and significantly suppressed virus shedding. In conclusion, although the H5N1 VLP vaccines induced lower HI antibody titres than the commercial vaccine, the H5N1 VLP vaccines induced homologous protection efficacy comparable to the commercial vaccine. These findings are consistent with previous study [7].

Adjuvants play a pivotal role in influenza vaccines to induce broad protection against homologous and heterologous viruses challenge. Studies have shown that the addition of adjuvants such as Matrix M, JVRS-100, and AS03 to virosomal H5N1 influenza vaccines induces broadly protective immune responses including cross-clade H5N1 neutralization against heterologous H5N1 viruses [30–32]. A study has shown that H5N1 subunit vaccine combined with adjuvant induces superior protection against H5N1 lethal challenge than non-adjuvant vaccine [17]. Study has also shown that adjuvant can enhance the cellular immune response of H5N1 subunit vaccines against homologous and heterologous H5N1 viruses challenge [23]. In the present study, we compared two adjuvants including ISA 201 and ISA 71, and both the adjuvanted H5N1 VLP vaccines provided insufficient protection against antigenically divergent H5N1-D889 virus challenge at low dose. Studies have shown that HI and neutralizing antibodies that target the highly variable globular head domain of influenza HA and are often strain specific [22,33]. Therefore, both the adjuvanted H5N1 VLP vaccines induced strain-restricted immune responses, that is, the strain specificity of vaccines mirrors the exquisite specificity of the HI and neutralizing antibodies. Due to the large difference in HA head between H5N1-SD57 and H5N1-D889 strains, both the adjuvanted H5N1 VLP vaccines induced robust HI and neutralizing antibodies against homologous H5N1-SD57 strain, and induce only low HI and neutralizing antibodies against antigenically divergent H5N1-D889 strain (Figure 4). Although the serological immunity induced by the two H5N1 VLP vaccines was no significant difference, ISA 71 adjuvanted H5N1 VLP vaccine provided protection with 90% survival of chickens significantly higher than the 40% survival rate provided by ISA 201 adjuvanted H5N1 VLP vaccine (Figure 4F). We hypothesized that cellular immunity and HA-associated immunity such as stalk-speciﬁc immunity induced by ISA 71 adjuvanted H5N1 VLP vaccine contributed to the increased protection against H5N1-D889 virus.

Cell-mediated immune responses induced by influenza vaccines have been proven to be a key factor in providing broad-spectrum protection against heterologous influenza viruses [10,23]. Our results indicated that ISA 71 adjuvanted H5N1 VLP vaccine induced significantly higher levels of Th1- and Th2-mediated T-cell immune responses than ISA 201 adjuvanted H5N1 VLP vaccine (Figure 3). The study has shown that influenza-specific Th1 immune responses contribute to cross-protection against heterologous influenza viruses challenge [34]. The increased Th1 and Th2 immune responses in the ISA 71 adjuvanted H5N1 VLP vaccine provided additional protection against H5N1-D889 virus challenge when serum antibodies alone are insufficient for protection. In addition, HA-associated stalk-specific immune responses may also play a pivotal role in conferring cross-protection against antigenically divergent H5N1 virus challenge. Influenza vaccines bearing HA can refocus immunity towards the highly conserved HA stalk domain to induce stalk-specific antibody responses that confer cross-protection against challenges with heterologous and heterosubtypic influenza viruses [35]. Therefore, multiple immune components including cellular immunity and potentially HA stalk-specific immunity elicited by the ISA 71 adjuvanted H5N1 VLP vaccine may contribute to cross-protection against antigenically divergent H5N1 virus challenge in the absence of effective HI and neutralizing antibodies.

Besides the selection of the appropriate adjuvant, the strategy of enhancing vaccine dose is another option to improve the efficacy of vaccines. Compared to the low-dose ISA 71 adjuvanted vaccine, the high-dose ISA 71 adjuvanted vaccine significantly improved HI antibodies against homologous H5N1 virus, moderately increased HI antibodies against antigenically divergent H5N1 virus, and also significantly improved neutralizing antibodies against antigenically divergent H5N1 virus (Figure 5). Compared to the commercial inactivated vaccine, the high-dose (80 μg) of ISA 71 adjuvanted vaccine induced significantly high levels of neutralizing antibodies against H5N1-D889 virus and conferred 100% sterilizing protection from antigenically divergent H5N1 challenge (Figure 6). In contrast to the HI antibody titres, the neutralizing antibody titres were antigen-dependent and more closely reflect the capability of H5N1 VLP vaccine to suppress virus shedding and provide heterologous protection. This is consistent with studies that HA-based influenza subunit vaccine induces neutralizing antibody responses against homologous and heterologous H5-subtype influenza viruses [36]. Study has also shown that neutralizing antibody titres are better predictors for protection than HI titres [37]. Broadly neutralizing antibodies can neutralize all representative H5N1 viruses and offer protection against H5N1 lethal challenge [38]. Study has shown that H5N1 inactivated vaccine has a poor capacity to induce neutralizing antibodies [39], which is consistent with our study. our results demonstrated that commercial inactivated vaccine induced low levels of HI and neutralizing antibodies and provided protection against antigenically divergent H5N1 challenge. This protection induced by commercial inactivated vaccine may be mediated by correlates with H5 cross-reactive antibodies in the absence of H5N1 HI and neutralizing antibodies [22]. Study has reported that the strategy of sequential immunization with chimeric HA-based vaccines redirects immunity toward the conserved stalk region to induce stalk-specific immune responses and provide heterologous protection against influenza viruses [40]. The strategy of enhancing vaccine dose may also redirect immunity toward the conserved stalk region to improve the stalk-specific immune response, which needs further study.

In this study, the H5N1 VLP vaccine required a large dose of antigen to achieve full protection against antigenically divergent H5N1 virus challenge, which needed further modification. Studies have shown that neuraminidase (NA) [41], nucleoprotein (NP) [20], inﬂuenza conserved epitopes such as HA-stalk, M2e [42], Toll-like receptor ligands [19,34], and immunological proteins adjuvants such as LTB [19] contribute to cross-protection against heterologous virus challenge. These immunological components can be incorporated into H5N1 VLP to enhance the cross-protection capacity of H5N1 VLP vaccines. Strategies to enhance the immunogenicity of HA can also be chosen to improve the immune responses of influenza VLP vaccines [43,44]. Strategies of construction of the multivalent VLP, mucosal immunization, and combined nanoparticle were demonstrated to enhance the cross-protective efficacy of influenza vaccines [23,45,46].

In conclusion, we developed an H5N1 VLP vaccine, which induced efficient serum antibody responses and protected against homologous H5N1 virus challenge in chickens. Choosing a more appropriate adjuvant and increasing the vaccine dose could further enhance the cross-protective efficacy of H5N1 VLP vaccine. The protection against antigenically divergent H5N1 virus is mediated by cellular immunity and neutralizing antibodies. Our results suggest that H5N1 VLP vaccine can induce cross-protection against homologous and antigenically divergent H5N1 virus challenge, which is determined by adjuvant and vaccine dose.

## Supplementary Material

Supplemental Material
