# Supplementary material for: A single immunization with H5N1 virus-like particle vaccine protects chickens against divergent H5N1 influenza viruses and vaccine efficacy is determined by adjuvant and dosage
Source: Emerg Microbes Infect. 2023 Nov 23;13(1):2287682. doi: 10.1080/22221751.2023.2287682 (PMC10763850; doi:10.1080/22221751.2023.2287682)

**Supplementary data**

**A single immunization with H5N1 virus-like particle vaccine protects chickens against divergent H5N1 influenza viruses and vaccine efficacy is determined by adjuvant and dosage**

Dexin Kong^1,2,3,4^, Yanjuan He^1,2,3,4^, Jiaxin Wang^1,2,3,4^, Lanyan Chi^1,2,3,4^, Xiang Ao^1,2,3,4^, Hejia Ye^5^, Weihong Qiu^5^, Xiutong Zhu^5^, Ming Liao^1,2,3,4^* and Huiying Fan^1,2,3,4^*

^1^College of Veterinary Medicine, South China Agricultural University, Guangzhou 510642, Guangzhou, China

^2^Key Laboratory of Zoonosis Prevention and Control of Guangdong Province, Guangzhou, China

^3^Key Laboratory of Veterinary Vaccine Innovation of the Ministry of Agriculture and Rural Affairs, Guangzhou, China

^4^National and Regional Joint Engineering Laboratory for Medicament of Zoonosis Prevention and Control, Guangzhou, China

^5^Guangzhou South China Biological Medicine Co., Ltd, Guangzhou, China

*Corresponding author:

Huiying Fan, College of Veterinary Medicine, South China Agricultural University, Guangzhou 510642, China. Tel/ Fax: 0086-20-85280240; Email: [fanhy@scau.edu.cn](mailto:fanhy@scau.edu.cn)

Ming Liao, College of Veterinary Medicine, South China Agricultural University, Guangzhou 510642, China. Tel/ Fax: 0086-20-85280240; E-mail: [mliao1968@163.com](mailto:fanhy@scau.edu.cn).

**Supplementary Table 1. Program of vaccination and challenge**

| Exp ^a^ | Antigen | Adjuvant | Doses (per) | Group (chickens) | Challenge virus | Challenge ^b^ |
| --- | --- | --- | --- | --- | --- | --- |
| 1 | PBS | - | 300 µL | 5 | H5N1-SD57 | Day 21 |
|  | H5N1 VLP | ISA 201 or ISA 71 | 40 µg in 300 µL | 10 |  | Day 21 |
|  | Commercial vaccine | - | 300 µL | 10 |  | Day 21 |
| 2 | PBS | - | 300 µL | 8 | H5N1-D889 | Day 21 |
|  | H5N1 VLP | ISA 201 | 40 µg in 300 µL | 13 |  | Day 21 |
|  | H5N1 VLP | ISA 71 | 40 µg in 300 µL | 13 |  | Day 21 |
| 3 | PBS | - | 300 µL | 5 | H5N1-D889 | Day 21 |
|  | H5N1 VLP | ISA 71 | 60 µg in 300 µL | 10 |  | Day 21 |
|  | H5N1 VLP | ISA 71 | 80 µg in 300 µL | 10 |  | Day 21 |
|  | Commercial vaccine | - | 300 µL | 10 |  | Day 21 |

^a^ Exp: Experiment

^b^ On Day 21, immunized chickens were intranasally challenged with the H5N1-SD57 virus or H5N1-D889 virus.

**Supplementary Table 2. Sequences of primers used for quantitative real-time PCR**

| Gene | Primer sequences (5′-3′) | Product size (bp) | Accession no. |
| --- | --- | --- | --- |
| IFN-γ | F: ACCTTCCTGATGGCGTGAAG | 102 | AJ634956.1 |
|  | R: TGAAGAGTTCATTCGCGGCT |  |  |
| IL-4 | F: ATGACATCCAGGGAGAGGTTT | 235 | GU119892.1 |
|  | R: ATTGGAGTAGTGTTGCCTGCT |  |  |
| IL-17 | F: ACAGGAGATCCTCGTCCTCC | 95 | AY744450.1 |
|  | R: TGACACATGTGCAGCCCAC |  |  |
| β-actin | F: TGGGTATGGAGTCCTGTGGT | 136 | NM_205518.1 |
|  | R: CTGTCAGCAATGCCAGGGTA |  |  |

**Supplementary Table 3. Virus shedding after a lethal dose homologous influenza virus challenge of chickens**

| Group | Challenge virus | 5 dpc ^a^ | | Total virus shedding number/total number | No. clinical symptoms | Survival /total |
| --- | --- | --- | --- | --- | --- | --- |
|  |  | Oropharyngeal swab | Cloacal swab |  |  |  |
| H5N1 VLP  +ISA 201 | SD57 | 0/10 | 0/10 | 0/10 | 0 | 10/10 |
| Commercial vaccine | SD57 | 0/10 | 0/10 | 0/10 | 0 | 10/10 |
| PBS | SD57 | NA ^b^ | NA | NA | 5 | 0/5 |
| H5N1 VLP  +ISA 71 | SD57 | 0/10 | 0/10 | 0/10 | 0 | 10/10 |
| Commercial vaccine | SD57 | 0/10 | 0/10 | 0/10 | 0 | 10/10 |
| PBS | SD57 | NA | NA | NA | 5 | 0/5 |

Note: SD57 is the virus of A/Chicken/Shandong/WFZC/2017(H5N1). The oropharyngeal and cloacal swab samples were collected at 5 days post-challenge. Virus positivity or shedding was determined by inoculating each swab solution into 3 eggs of 10-day-old specific-pathogen-free chicken embryos. ^a^dpc, days post-challenge. ^b^ NA, not applicable due to the death of chickens.

**Legend for supplementary figure**

**Figure S1. Phylogenic tree of H5N1 influenza viruses using HA gene sequences with available the other influenza viruses.**

The H5Nx viruses of clade 2.3.2 and 2.3.4.4, including our H5N1 viruses were used to perform the phylogenic analysis. Neighbor-joining (NJ) phylogenetic trees for the codon alignment of the HA gene segments were estimated using MEGA (version 7) program. Node support was determined by nonparametric bootstrapping with 1,000 replicates. The phylogenetic tree was visualized in the FigTree (version 1.4.3) program. The H5N1 viruses used in this study were labeled in red.

**Figure S2. The induction of cytokines** **between virus stimulation and antigen stimulation (n=3).**

(A) The mRNA expression levels of IFN-γ between virus stimulation and antigen stimulation. (B) The mRNA expression levels of IL-4 between virus stimulation and antigen stimulation. (C) The mRNA expression levels of IL-17 between virus stimulation and antigen stimulation. The data were analyzed statistically using an unpaired t-test. Data represented mean ± SD. Statistically significant differences are indicated by * p < 0.05.

**Figure S1**

**
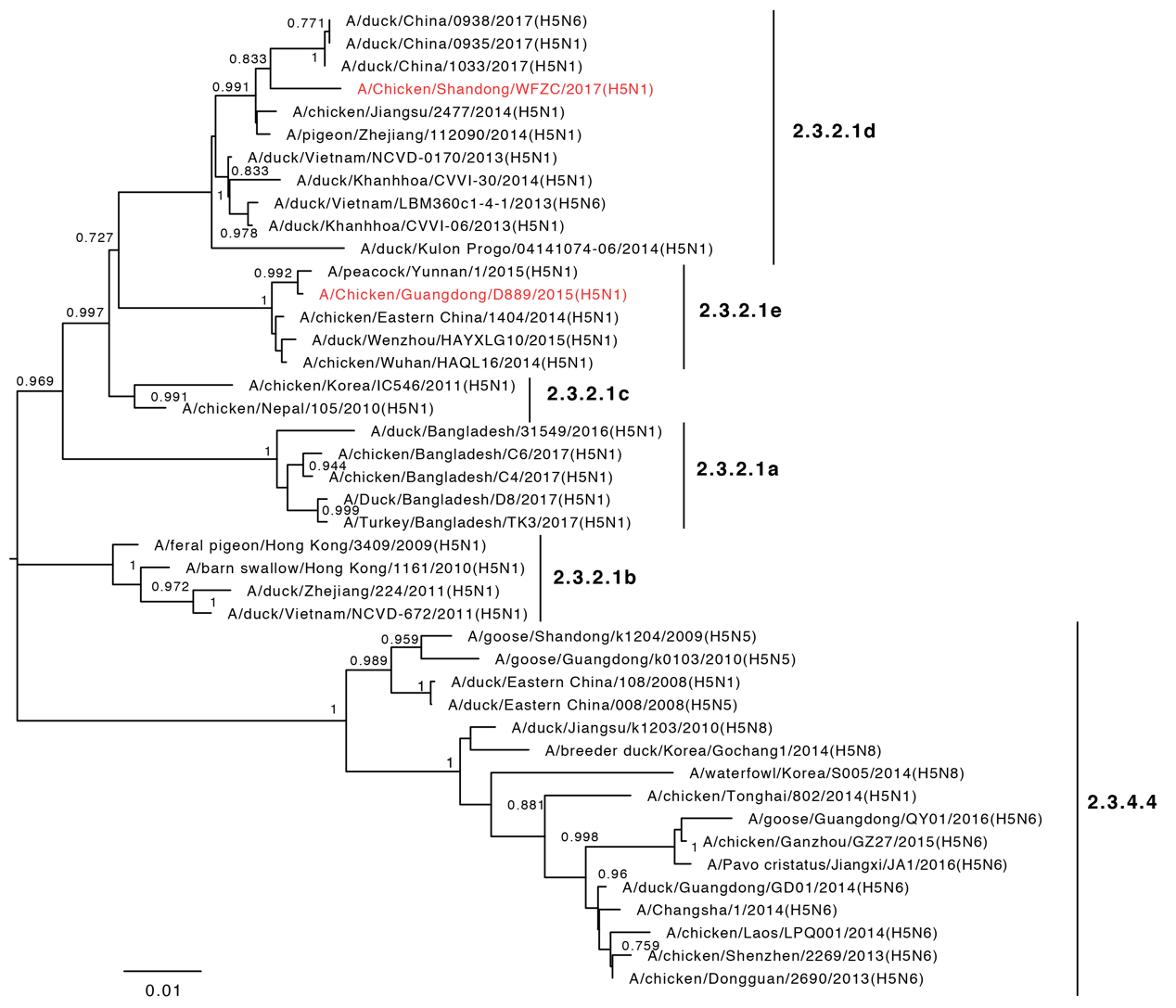
**

**Figure S2**


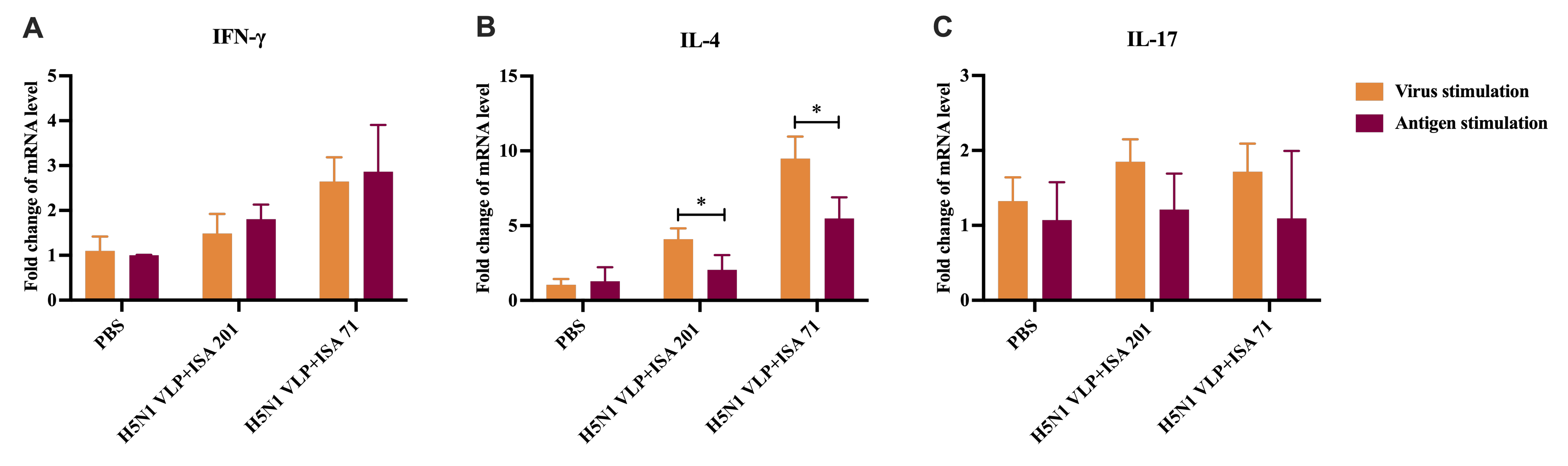

Supplement: Supplemental Material [file TEMI_A_2287682_SM5942.docx]
